# Efficacy and safety of a non-immersive virtual reality-based neuropsychological intervention for cognitive stimulation and relaxation in patients with critical illness: study protocol of a randomized clinical trial (RGS-ICU)

**DOI:** 10.1186/s12888-024-06360-4

**Published:** 2024-12-18

**Authors:** Marta Godoy-González, Josefina López-Aguilar, Sol Fernández-Gonzalo, Gemma Gomà, Lluís Blanch, Santiago Brandi, Sergi Ramírez, Joel Blasi, Paul Verschure, Gemma Rialp, Miquel Roca, Margalida Gili, Mercè Jodar, Guillem Navarra-Ventura

**Affiliations:** 1 Critical Care Department, Parc Taulí Hospital Universitari, Institut d’Investigació i Innovació Parc Taulí (I3PT-CERCA), Universitat Autònoma de Barcelona, Sabadell, Spain; 2 Department of Clinical and Health Psychology, Universitat Autònoma de Barcelona, Bellaterra, Spain; 3 Centro de Investigación Biomédica en Red de Enfermedades Respiratorias (CIBERES), Instituto de Salud Carlos III, Madrid, Spain; 4 Centro de Investigación Biomédica en Red de Salud Mental (CIBERSAM), Instituto de Salud Carlos III, Madrid, Spain; 5 Eodyne Systems S.L, Barcelona, Spain; 6 CSIC Alicante Institute of Neuroscience and Department of Health Psychology, Universidad Miguel Hernández de Elche - UMH, Elche, Spain; 7 Critical Care Department, Hospital Universitari Son Llàtzer, Palma, Spain; 8 Department of Medicine, University of the Balearic Islands (UIB), Palma, Spain; 9 Health Research Institute of the Balearic Islands (IdISBa), Hospital Universitari Son Espases, Palma, Spain; 10 Research Institute of Health Sciences (IUNICS), University of the Balearic Islands (UIB), Palma, Spain; 11 Department of Psychology, University of the Balearic Islands (UIB), Palma, Spain; 12 Neurology Department, Parc Taulí Hospital Universitari, Institut d’Investigació i Innovació Parc Taulí (I3PT-CERCA), Universitat Autònoma de Barcelona, Sabadell, Spain

**Keywords:** Cognition, Comfort, Critical care, Critical illness, Intensive care unit, Mental health, Post-intensive care syndrome, Prevention, Rehabilitation, Relaxation

## Abstract

**Background:**

Experiencing a critical illness may be a stressful life event that is also associated with cognitive dysfunction during and after the intensive care unit (ICU) stay. A deep-tech solution based on non-immersive virtual reality, gamification and motion capture called Rehabilitation Gaming System for Intensive Care Units (RGS-ICU) has been developed that includes both cognitive stimulation and relaxation protocols specifically designed for patients with critical illness. This study aims to evaluate whether the cognitive and relaxation protocols of the RGS-ICU platform are 1) effective in improving neuropsychological outcomes during and after ICU stay and 2) safe for patients with critical illness.

**Methods:**

This is a study protocol for a multicenter longitudinal randomized clinical trial. At least 80 patients with critical illness will be included: 40 experimental subjects and 40 control subjects. Patients in the experimental group will receive daily 20-min sessions of cognitive stimulation and relaxation with the RGS-ICU platform adjuvant to standard ICU care in their own rooms during the ICU stay and until discharge from the ICU or up to a maximum of 28 days after randomization, provided they are alert and calm. Patients in the experimental group will be constantly monitored as part of standard ICU care to ensure the safety of the intervention and that no avoidable adverse events occur. Patients in the control group will receive standard ICU care. The primary outcome is objective cognition 12 months after ICU discharge, assessed with a composite index including measures of attention, working memory, learning/memory, executive function and processing speed. The secondary outcome is the safety of the intervention, assessed by considering the number of sessions terminated early due to unsafe events in physiological parameters. Other outcomes are comfort experienced during the ICU stay, and subjective cognition, mental health (anxiety, depression and post-traumatic stress disorder), functionality and health-related quality of life 12 months after ICU discharge.

**Discussion:**

The expected results are 1) better neuropsychological outcomes during and after the ICU stay in patients in the experimental group compared to patients in the control group and 2) that the cognitive and relaxation protocols of the RGS-ICU platform are safe for patients with critical illness.

**Trial registration:**

Clinicaltrials.gov NCT06267911. Registered on February 20, 2024.

**Supplementary Information:**

The online version contains supplementary material available at 10.1186/s12888-024-06360-4.

## Introduction

### Background and rationale

Admission to an intensive care unit (ICU) is a potentially traumatic experience, especially for patients who are mentally vulnerable. Beyond the stress and anxiety associated with the ICU environment and medical procedures, survivors of critical illness are at risk of developing cognitive and psychological sequelae related to post-intensive care syndrome (PICS) [1, 2]. These sequelae can persist for months or even years after ICU discharge, compromise patients’ functionality and health-related quality of life (HRQoL) [3, 4], and carry high personal, social, medical and economic costs [5].

Cognitive deficits related to PICS, whether objective or subjective, typically affect attention, working memory, learning/memory, executive function and processing speed [6, 7]. Psychological disturbances often include anxiety, depression and post-traumatic stress disorder (PTSD) as the most common mental health manifestations [8, 9]. The main risk factors associated with these sequelae are delirium, invasive mechanical ventilation (MV) and prolonged deep sedation during ICU stay [10–12]. In contrast, cognitive reserve (i.e., the patient's brain's resistance to neurological damage) may be a protective factor [6, 7, 13, 14].

During the ICU stay, patients with critical illness spend most of the day bedridden, may have verbal communication problems and develop ICU-acquired muscle weakness [15]. To date, several e-health solutions have been proposed to cognitively stimulate and help relax these patients, but few studies have examined whether these types of interventions are effective in improving neuropsychological outcomes during and after the ICU stay and safe for patients with critical illness [16–18].

One such e-health solution is the Early Neurocognitive Rehabilitation in Intensive Care (ENRIC) platform. To the authors’ knowledge, this was the first non-immersive virtual reality (VR)-based cognitive intervention designed and developed specifically for patients with critical illness and the ICU environment (Clinicaltrials.gov Identifier: NCT02078206). In a first proof-of-concept (PoC) study (*n* = 20), Turon et al. [19] demonstrated the feasibility, safety, tolerability and potential efficacy of this intervention to stimulate the brain during ICU stay using a surrogate measure of cognitive function (i.e., heart rate variability, HRV). In a second randomized clinical pilot (*n* = 42, 21 experimental subjects and 21 control subjects), Navarra-Ventura et al. [13] demonstrated that cognitive stimulation with the ENRIC platform improved working memory performance shortly after ICU discharge and that this advantage was maintained in the long term, in line with results previously obtained using HRV. A trend toward reduced rates of anxiety and depression was also observed, although it did not reach statistical significance. However, this study was limited by an intervention that included only cognitive protocols but no specific relaxation content and by the high dropout rate related to the Covid-19 pandemic situation, with only 10 experimental subjects and 14 control subjects completing all post-ICU follow-up visits. Interestingly, in another PoC and pilot study (*n* = 20), Martí-Hereu et al. [20] demonstrated that watching videos of relaxing experiences using immersive VR goggles is feasible, tolerable and can help reduce anxiety during ICU stay in patients with critical illness.

Based on these and other experiences in stroke neurorehabilitation [21], a specific solution for patients with critical illness called Rehabilitation Gaming System for Intensive Care Units (RGS-ICU) was designed and developed that includes both cognitive stimulation protocols modeled after the ENRIC platform exercises and relaxing videos set in nature and fantasy landscapes to promote stress and anxiety reduction in patients with critical illness.

The following is the study protocol of a randomized clinical trial (RCT) using the RGS-ICU platform whose overall objective is to evaluate the efficacy and safety of cognitive stimulation and relaxation as an adjunct to standard ICU care (experimental condition) compared to standard ICU care alone (control condition).

### Objectives and hypotheses

The main objective is to assess whether patients in the intervention group show better objective cognitive performance than patients in the control group three and 12 months after ICU discharge. The secondary objective is to assess whether the cognitive and relaxation protocols of the RGS-ICU platform are safe for patients with critical illness. Other objectives are to explore whether patients in the intervention group show greater comfort during the ICU stay, as well as better outcomes in subjective cognition, mental health (anxiety, depression and PTSD), functionality and HRQoL than patients in the control group three and 12 months after ICU discharge.

The main hypothesis is that patients in the intervention group will show better objective cognitive performance than patients in the control group three and 12 months after ICU discharge. The secondary hypothesis is that the cognitive and relaxation protocols of the RGS-ICU platform will be safe for patients with critical illness. Other hypotheses are that patients in the intervention group will show greater comfort during the ICU stay, as well as better outcomes in subjective cognition, mental health (anxiety, depression and PTSD), functionality and HRQoL than patients in the control group three and 12 months after ICU discharge.

### Trial design

A two-arm, parallel group, open-label, superiority RCT with a 1:1 allocation ratio has been designed to evaluate the efficacy and safety of receiving cognitive stimulation and relaxation as an adjunct to standard ICU care (experimental [RGS-ICU] condition) compared with receiving standard ICU care alone (control [treatment as usual, TAU] condition).

## Methods

The Standard Protocol Items: Recommendations for Interventional Trials (SPIRIT) reporting guidelines have been used to write this study protocol (Additional file 1) [22].

### Study setting

Four centers are participating in this RCT: the Parc Taulí University Hospital and the Parc Taulí Research and Innovation Institute in Sabadell, Barcelona (Catalonia), and the Son Llàtzer University Hospital and the University of the Balearic Islands-Research Institute of Health Sciences in Palma, Mallorca (Balearic Islands), Spain.

The Parc Taulí and Son Llàtzer University Hospitals have a total of 57 medical-surgical ICU beds between the two centers, both in single (*n* = 50 beds) and shared (*n* = 7 beds) rooms, which admit about 1,400 patients with critical illnesses each year, of whom 300 patients are expected to be candidates for participation in the study.

### Eligibility criteria

Included will be 1) adult patients (≥ 18 years); 2) admitted to a medical-surgical ICU; 3) for respiratory failure, cardiogenic shock or septic shock; 4) with an expected ICU stay of ≥ 48 h; 5) resident in Catalonia or the Balearic Islands; 6) who speak Catalan and/or Spanish; and 7) who can give consent on their own or through an authorized representative (e.g., a family member).

Patients will be excluded if they have 1) a history of intellectual disability or other neurodevelopmental disorders, such as autism spectrum disorder or attention deficit/hyperactivity disorder; 2) a history of neurological disorder, dementia or other neurodegenerative disease, such as epilepsy, Alzheimer's disease, Parkinson's disease or multiple sclerosis; 3) a history of brain damage, such as traumatic brain injury or stroke; 4) history of severe psychiatric illness, such as psychotic, bipolar, depressive, obsessive–compulsive, post-traumatic or personality disorder; 5) suspected or confirmed substance use disorder; 6) suspected or confirmed communicable disease in an isolated patient; 7) uncorrected hearing or visual impairment; 8) any medical condition that prevents safe upper extremity mobility, such as skin lesions, burns or fractures; 9) life expectancy < 12 months; or 10) are enrolled in another RCT that does not allow co-enrollment.

### Intervention

The main objective of the intervention is to provide cognitive stimulation and relaxation specifically tailored to the needs of patients with critical illness and the characteristics of the ICU environment, fostering motivation and engagement with the sessions, regardless of the accuracy of the responses. To improve adherence, the cognitive and relaxation protocols of the RGS-ICU platform are based on non-immersive VR, gamification and motion capture to reduce monotony and boredom during sessions and avoid frustration.

#### Characteristics of the RGS-ICU platform

**Fig. 1:**
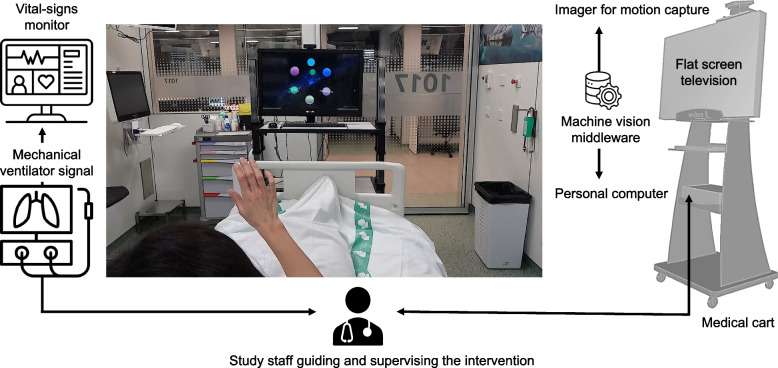
Schematic diagram of the RGS-ICU platform, bedside monitor and ventilator. RGS-ICU, Rehabilitation Gaming System for Intensive Care Units

The RGS-ICU platform has been designed at the Parc Taulí Research and Innovation Institute with the collaboration of the University of the Balearic Islands-Research Institute of Health Sciences by a multidisciplinary team of (neuro)psychologists, psychiatrists, intensivists, nurses, and other biomedical researchers, and has been developed by a group of software engineers from Eodyne Systems S.L., a company dedicated to the development of science-based solutions for intervention, monitoring, diagnosis and prognosis in brain health. It consists of a personal computer, a flat-screen TV and an imager for motion capture placed on a medical cart for easy transfer to each ICU bed. The system captures and interprets the patient's upper extremity movements using machine vision middleware that connects these movements to multimedia content for cognitive stimulation and relaxation. This approach to patient-system interaction avoids the risk of cross-infection inherent in sharing physical devices such as touch screens or game controllers. Although patients do not have physical contact with any component of the RGS-ICU platform at any time, for infection control reasons, it can be easily cleaned and disinfected after each session (Fig. 1 and Additional file 2).

The RGS-ICU platform is switched on when plugged in to a power source. To adapt the system to the patient's upper extremity mobility capabilities, it is necessary to calibrate the motion sensor and algorithm and adjust the distance between the patient and the webcam before each session of cognitive stimulation. To do this, the patient will be asked to raise one of his or her upper extremities against gravity in front of the webcam sensor at an angle of 45 to 90 degrees to the body to calibrate motion detection and recognition. Occasionally, the patient’s ICU bed will need to be raised or lowered. Bed adjustments and calibration of the motion capture system take less than 2 min. Relaxation protocols are passive exercises in which patients do not have to move their upper extremities and therefore do not require motion sensor calibration.

**Fig. 2:**
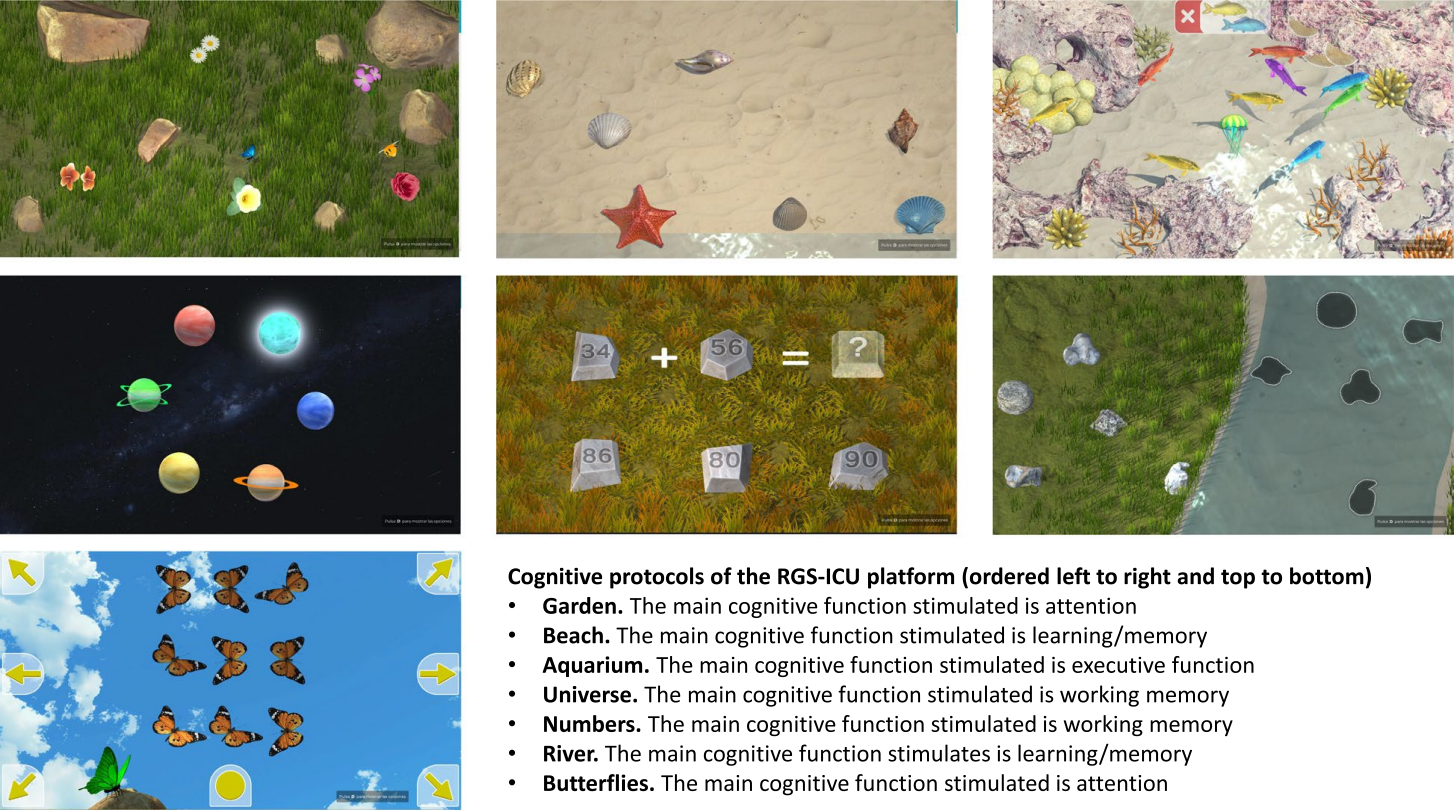
Examples of gamified cognitive protocols of the RGS-ICU platform. RGS-ICU, Rehabilitation Gaming System for Intensive Care Units

**Fig. 3:**
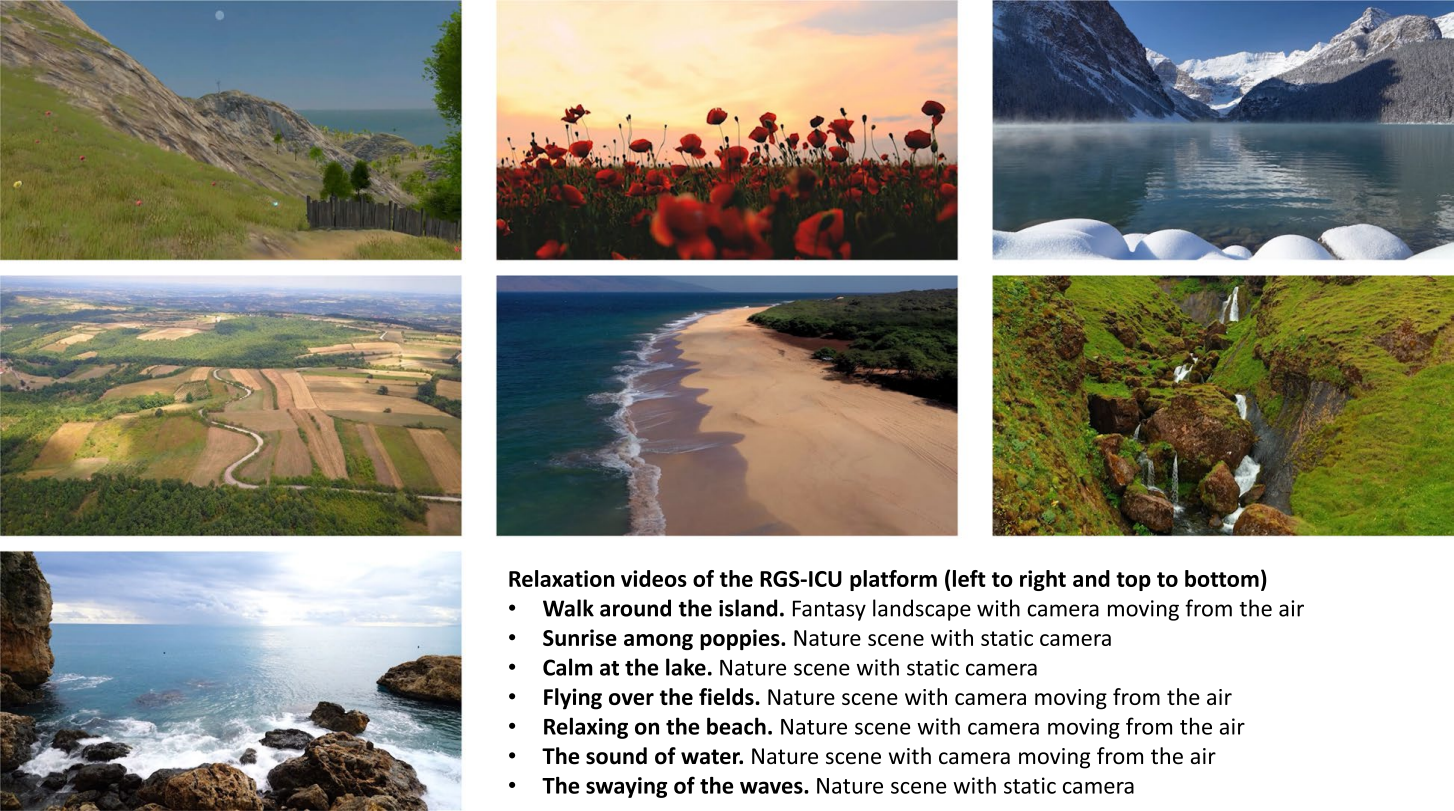
Examples of relaxing protocols of the RGS-ICU platform. RGS-ICU, Rehabilitation Gaming System for Intensive Care Units

The RGS-ICU platform includes seven gamified cognitive protocols with six levels of difficulty, each of them specifically designed to stimulate specific cognitive domains: attention (“Garden” and “Butterflies”), working memory (“Universe” and “Numbers”), learning/memory (“Beach” and “River”), and executive function (“Aquarium”), whereas processing speed is cross stimulated in all protocols (Fig. 2). It also includes seven videos with relaxing music and nature sounds to reduce stress and anxiety related to the ICU stay. Of these, six videos are set in natural environments, either with the camera static (“Sunrise among poppies”, “Calm at the lake” and “The swaying of the waves”) or moving from the air (“Flying over the fields”, “Relaxing on the beach” and “The sound of water”), while the seventh video is set on a fantasy island and simulates a relaxing walk (“Walk around the island”) (Fig. 3). Finally, an infographic on the ICU environment and the characteristics of the cognitive, mental and physical state of patients with critical illness is included for psychoeducational purposes.

Additional file 3 is a video with four examples of the gamified cognitive protocols of the RGS-ICU platform. Additional file 4 includes usability and satisfaction data with the RGS-ICU platform from a group of 15 patients with critical illness.

#### Experimental condition: RGS-ICU group

Patients assigned to this arm will receive flexible cognitive stimulation and relaxation sessions in addition to standard ICU care every day between 9 a.m. and 1 p.m. in their own rooms if they score ≥ 13 on the Glasgow Coma Scale (GCS) [23] and between -1 and + 1 on the Richmond Agitation-Sedation Scale (RASS) [24]. Delirium (score ≥ 3 on the Confusion Assessment Method for the ICU-7, CAM-ICU-7) in cooperative patients capable of following simple commands will not preclude participation in sessions [25–27]. All sessions will be guided and supervised by trained study staff, including research (neuro)psychologists and clinical staff, when medical, nursing or room cleaning procedures are not being performed.

**Fig. 4:**
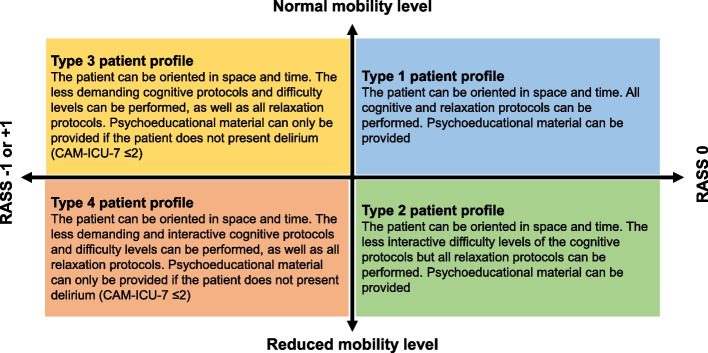
Types of patient profile according to RASS, CAM-ICU-7 and mobility level. RASS, Richmond Agitation-Sedation Scale; CAM-ICU-7, Confusion Assessment Method for the Intensive Care Unit-7. Patients with a normal level of mobility lift and hold at least one upper extremity straight against gravity without difficulty. Patients with a reduced level of mobility lift and hold the upper extremities straight against gravity with difficulty

The type and difficulty levels of the cognitive stimulation and relaxation protocols included in each session will be determined by the patient's ability to interact with the RGS-ICU platform, assessed daily at the beginning of the session considering mental status (consciousness [GCS], sedation/alertness [RASS] and delirium [CAM-ICU-7]) and mobility level (ability to lift and hold at least one upper extremity straight against gravity). To adapt the intervention to these variables, different types of interaction and workload have been established, which are summarized in four types of patient profiles (Table 1 and Fig. 4).

**Table 1:** Patient profile according to mental status and mobility level

Mental status			Mobility level	Patient profile
**GCS score**	**CAM-ICU-7 score**	**RASS score**		
13–15	Negative (0–2)	0	Normal^a^	Type 1^c^
			Reduced^b^	Type 2^d^
		-1 or + 1	Normal^a^	Type 3^e^
			Reduced^b^	Type 4^f^
	Positive (3–7)	-1 to + 1	Normal^a^	Type 3^e^
			Reduced^b^	Type 4^f^

*GCS* Glasgow Come Scale, *CAM-ICU-7* Confusion Assessment Method for the Intensive Care Unit-7, *RASS* Richmond Agitation-Sedation Scale

^a^Patient lifts and holds at least one upper extremity straight against gravity without difficulty

^b^Patient lifts and holds upper extremities straight against gravity with difficulty

^c^Maximum interaction and high level of cognitive demand

^d^Minimal interaction and high level of cognitive demand

^e^Moderate interaction and low level of cognitive demand

^f^Minimal interaction and low level of cognitive demand

Although the intervention may vary according to the type of patient profile, sessions have been standardized as follows: the average duration of a session will be 15–20 min, but patients will be encouraged to continue up to a maximum of 30 min if adequately tolerated. At the beginning of each session, patients will be oriented in space and time (about 1 min). Then, up to a maximum of four cognitive protocols (about 5 min each) will be applied to stimulate attention, working memory, learning/memory and/or executive function (in that order), as appropriate. By default, progression from easier to more difficult levels within a cognitive protocol will be made after successfully passing the previous level, so that patients who complete a portion of the exercises will be able to start the next session from the last level completed without errors. Preferably after cognitive stimulation, one or more relaxing videos will be shown to help reduce stress and anxiety (up to 10 min). Finally, general psychoeducational information about the ICU environment and the characteristics of the cognitive, mental and physical status of patients with critical illness will also be provided (about 5 min). By default, this information will be provided only once on the first day that the patient does not present delirium (score ≤ 2 on the CAM-ICU-7) but will be repeated as many times as necessary in subsequent sessions if requested by the patient or family members. Due to changes in mental status and mobility level and medical complications that may arise during the ICU stay, the study staff administering and supervising the intervention will also be able to choose which specific cognitive and relaxation protocols and difficulty levels are administered in each session, always within the parameters of each patient’s profile. The patient’s needs and preferences (e.g., the best time in the morning to apply the intervention, the cognitive and relaxation protocols to be administered) will also be considered, if they are safe and compatible with the patient's profile.

Patients will be constantly monitored during the intervention as part of standard ICU care. This will provide information on changes in mental status, mobility level and physiological parameters (e.g., peripheral oxygen saturation [SpO2], respiratory rate [RR] and heart rate [HR]), allowing the study staff to adapt the interaction, workload and duration of each session to the type of patient profile in real time. Adverse events (e.g., unsafe physiological parameters, unintentional removal of catheters, endotracheal tubes or tracheostomy cannulas) and tolerability issues (e.g., fatigue, extreme sleepiness, confusion) during the intervention will result in interruption or early termination of the session. Interruption or early termination of a session will not preclude participation in subsequent sessions if safety criteria are met (see “Safety and tolerability data” and “Harms and adverse event reporting” for details).

The intervention will be discontinued if patients voluntarily withdraw consent or, if they have not yet been able to give consent themselves, their authorized representative voluntarily withdraws consent, requests to stop receiving the intervention, are transferred to another ICU or die during ICU stay. At the discretion of the clinical staff, the intervention will also be discontinued if there are changes in the patient's medical condition, such as worsening disease, that make continued participation inadvisable or if there are safety concerns that poses a risk to participants. In the event of discontinuation of the intervention, patients will continue to receive standard ICU care. Participants should not participate in other RCT intended to stimulate cognition and provide relaxation during or after the ICU stay.

#### Control condition: TAU group

Patients assigned to this arm will receive standard ICU care and should not participate in other RCT aimed at stimulating cognition and providing relaxation during or after the ICU stay.

Standard ICU care for RGS-ICU and TAU groups is based on the ABCDEF bundle, a set of recommendations to optimize recovery and outcomes in patients with critical illness [28, 29].

### Outcomes

The primary outcome is objective cognition 12 months after ICU discharge assessed with a global cognition index (z-score: mean = 0, standard deviation = 1) established on the basis of previous studies [6–8, 13, 14] and composed of validated neuropsychological measures of attention (Forward Digit Span subtest of the Wechsler Adult Intelligence Scale-IV, WAIS-IV), working memory (Backward Digit Span subtest of the WAIS-IV), learning/memory (Rey Auditory Verbal Learning Test, RAVLT), executive function (Trail Making Test, TMT; Stroop Color and Word Test, SCWT; FAS verbal phonemic fluency test) and processing speed (Digit Symbol-Coding subtest of the WAIS-IV; Symbol Digit Modalities Test, SDMT) [30–35].

The secondary outcome is the safety of the intervention assessed by considering the number of sessions terminated early due to unsafe events on physiological parameters [19].

Other outcomes are comfort experienced during ICU stay assessed at ICU discharge with the Patient Evaluation of Emotional Comfort Experienced questionnaire (PEECE) (range 0–48) [36], subjective cognition 12 months after ICU discharge assessed with the Perceived Deficits Questionaire-5 (PDQ-5) (range 0–20) [37, 38], anxiety 12 months after ICU discharge assessed with the Generalized Anxiety Disorder-7 questionnaire (GAD-7) (range 0–21) [39, 40], depression 12 months after ICU discharge assessed with the Patient Health Questionnaire-9 (PHQ-9) (range 0–27) [41, 42], PTSD 12 months after ICU discharge assessed with the Treatment-Outcome Post-Traumatic Stress Disorder Scale (TOP-8) (range 0–32) [43, 44], functionality 12 months after ICU discharge assessed with the World Health Organization Disability Assessment Schedule 2.0 (WHODAS 2.0) (range 0–100) [45], and HRQoL 12 months after ICU discharge assessed with the Short-Form Health Survey (SF-12) (range 0–100) [46, 47].

For all outcomes, except for intervention safety which is only evaluated in the RGS-ICU group, the difference between the RGS-ICU and TAU groups will be considered.

### Participant timeline

**Fig. 5:**
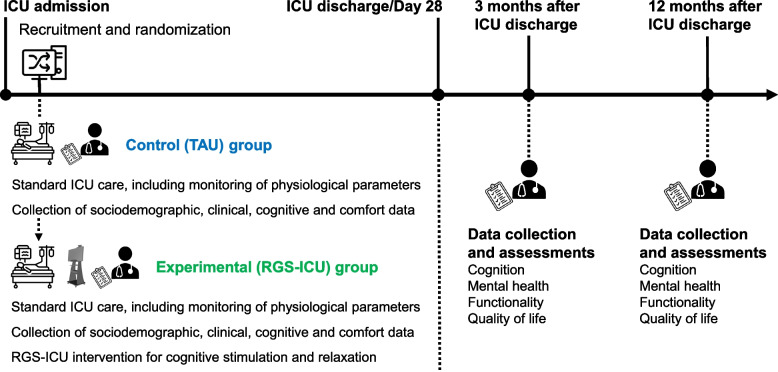
Timeline of study procedures. ICU, intensive care unit; TAU, treatment as usual; RGS-ICU, Rehabilitation Gaming System for Intensive Care Units

The planned duration of the study is 24 months, starting in the summer of 2024. Patients will be enrolled consecutively during the first 12 months. The intervention will be administered daily during the ICU stay, as long as the patient's medical condition permits, and will end at ICU discharge or a maximum of 28 days after randomization, whichever occurs first. All participants will receive standard ICU care. Follow-up visits will be performed three and 12 months after ICU discharge (Fig. 5).

### Sample size and power calculation

Sample size is based on the test of equality of two independent means for the primary outcome (objective cognition 12 months after ICU discharge) calculated using G-Power v3.1.9.7 software [48, 49]. Based on data from a randomized clinical pilot using the ENRIC platform [13], an eta squared effect size of 0.14 is anticipated. With a statistical power of 0.8 and a two-sided alpha level of 0.05, a minimum sample of 26 participants per group is needed to detect a Cohen's d effect size of 0.8 (total *n* = 52). Considering a dropout rate of 50% (15% during the ICU stay and 35% after ICU discharge), the minimum recruited sample needed for this RCT is 78 participants (*n* = 39 per group). Thus, at least 80 patients with critical illness (*n* = 40 per group) will be recruited, half of them in each participating hospital. However, since this RCT takes place in the ICU, it is to be expected that some dropouts will occur for reasons unrelated to the study itself (e.g., changes in health status that make continued participation inadvisable, mortality in the ICU, transfer to another ICU or withdrawal of consent previously given by an authorized representative). Thus, to maintain a sample size with sufficient power, these participants, estimated at 15%, will be replaced by new enrolments in a 1:1 ratio.

### Recruitment

Patients admitted to the ICU will be examined daily during the ICU stay, and, if they meet the eligibility criteria, they or their authorized representative(s) will be contacted for consent. If consent is initially obtained from an authorized representative (e.g., in the case of a comatose, deeply sedated or delirious patient), consent will be ratified once the patient is mentally competent, defined as GCS ≥ 13, RASS between -1 and + 1, and CAM-ICU-7 ≤ 2. Patients who refuse to participate, those whose authorized representatives refuse to participate, and those who refuse to participate even though their authorized representatives have given prior consent will be excluded.

### Allocation and blinding

After initial consent, patients will be assigned to one of the two study arms using the Research Electronic Data Capture (REDCap) randomization module. A blinded study coordinator, who will not be involved in patient care, intervention, data collection or assessments, will manage this computer-generated procedure. Due to the characteristics of the intervention, neither other study staff nor patients will be blinded. Data collection and assessments will also not be blinded. However, an independent statistician will perform data analysis in a blinded fashion.

### ICU data collection and assessments

The following data will be collected and assessed from ICU admission to ICU discharge or up to a maximum of 28 days after randomization by trained study staff. Collection and assessment of outcome measures for each patient will be performed by the same investigator throughout the study. If necessary, electronic medical records will be reviewed. Since patients will be admitted to the ICU, no retention problems are expected (Table 2).

**Table 2:** Schedule of study procedures during the ICU stay

	**Day** **0**	**Day** **1**	**Day** **2**	**Day** **…**	**Day 28/ICU discharge**
**Enrollment**					
Eligibility screening	x				
Informed consent	x				
Allocation	x				
**Intervention (RGS-ICU group only)**					
Cognitive stimulation and relaxation		x	x	x	x
Mobility level of the upper extremities^a^		x	x	x	x
Physiological parameters (SpO2, RR, HR)^b^		x	x	x	x
**ICU data collection and assessments**					
Age at ICU admission		x			
Sex at ICU admission		x			
Education at ICU admission		x			
Reason for admission (principal diagnosis)		x			
Medical comorbidities at ICU admission (CCI)		x			
Severity of illness at ICU admission (SAPS 3, APACHE)		x			
Organ failure at ICU admission (SOFA)		x			
Mental status (GCS, RASS, CAM-ICU-7)		x	x	x	x
Ventilatory support (if any)		x	x	x	x
Weaning failure (if on MV)		x	x	x	x
Pharmacological treatment (type and dose in milligrams/day)		x	x	x	x
Dyspnea, pain, worry and sadness (VAS)^c^		x	x	x	x
Experienced comfort (PEECE)					x
Cognitive screening (MoCA)					x

*ICU* Intensive Care Unit, *RGS-ICU* Rehabilitation Gaming System for Intensive Care Units, *CCI* Charlson Comorbidity Index, *SAPS 3* Simplified Acute Physiology Score 3, *APACHE II* Acute Physiology and Chronic Health Evaluation II; SOFA, Sequential Organ Failure Assessment, *SpO2* Peripheral oxygen saturation, *RR* Respiratory rate, *HR* Heart rate, *GCS* Glasgow Coma Scale, *RASS* Richmond Agitation-Sedation Scale, *CAM-ICU-7* Confusion Assessment Method for the ICU-7, *MV* Mechanical ventilation, *VAS* Visual Analogue Scale, *PEECE* Patient Evaluation of Emotional Comfort Experienced, *MoCA* Montreal Cognitive Assessment

^a^Ability to lift and hold at least one upper extremity straight against gravity

^b^Continuous variables automatically monitored as part of standard ICU care to ensure the safety of the intervention

^c^Assessed once daily in patients assigned to the control (treatment as usual, TAU) group and before and after each session in patients assigned to the experimental (RGS-ICU) group, provided the patient is mentally competent

#### Sociodemographic and clinical data

Age, sex, education, reason for admission (principal diagnosis), medical comorbidities (Charlson Comorbidity Index) [50], severity of illness (Simplified Acute Physiology Score 3 and Acute Physiology and Chronic Health Evaluation II) [51–54] and organ failure (Sequential Organ Failure Assessment, SOFA) [55] at ICU admission will be collected. Sequential data including mental status (GCS, RASS and CAM-ICU-7), mobility level (RGS-ICU group only), ventilatory support (none, non-invasive or invasive), weaning failure (if on MV) and pharmacological treatment (type and dose in milligrams/days) will be collected daily. Dyspnea, pain, worry and sadness will also be assessed once a day by visual analogue scales (VAS) with a range from 0 to 10 in patients assigned to the TAU group and before and after each intervention session in patients assigned to the RGS-ICU group, provided they are mentally competent.

#### Safety and tolerability data

The safety of the intervention will be assessed by considering the number of sessions terminated early due to unsafe events in physiological parameters, analyzing SpO2, RR and HR [19]. The safety analysis will be based on expert consensus criteria for unsafe active mobilization of patients with critical illness on MV: SpO2 < 90%, RR > 35 breaths/minute and HR > 150 beats/minute, all during ≥ 5 min [56]. Out-of-range values at the start of the session or changes > 20% from baseline in any physiological parameter will also be considered unsafe events requiring that the session not be started or terminated, respectively. Failure to start a session or early termination of a session will not preclude participation in subsequent sessions if the safety criteria are met. Safety physiological parameters will be automatically displayed on bedside monitors using advanced continuous monitoring systems as part of standard ICU care. An alarm will be triggered if an unsafe event occurs. Study staff guiding and supervising the sessions will also visually check the stability of physiological parameters to ensure the safety of the intervention and that no avoidable adverse events (e.g., unintentional removal of catheters, endotracheal tubs or tracheostomy cannulas) occur.

The tolerability of the intervention will be assessed by considering the number of completed sessions and the patients' tolerance to the interaction and workload of the cognitive and relaxation protocols. Therefore, the number of interruptions, the cause of the interruption (e.g., fatigue, extreme sleepiness, confusion), as well as the type and difficulty level of the cognitive and relaxation protocols in which the interruption occurred will be recorded.

#### Cognitive screening and experienced comfort data

On the day of discharge from the ICU, all participants will be administered the PEECE, a 12-item self-report questionnaire assessing subjective psychological well-being (Cronbach’s alpha = 0.74–0.88) [36]. The Montreal Cognitive Assessment (MoCA) test, a validated cognitive screening in survivors of critical illness, will also be administered [57, 58]. These data will only be collected if the patient is mentally competent.

#### Post-ICU assessments

The following data will be assessed three and 12 months after ICU discharge by trained study staff. Patient assessments after ICU discharge will be performed by the same investigator who followed them during their ICU stay. To promote retention, patients will be contacted by telephone to personally schedule follow-up visits. If the patient does not keep the first appointment, a second visit will be scheduled (Table 3).

**Table 3:** Schedule of study procedures after the ICU stay

	**3 months after ICU discharge**	**12 months after ICU discharge**
**Post-ICU assessments**		
Cognitive reserve (CRQ)^a^	x	
Estimated IQ (NART)^a^	x	
Cognitive screening (MoCA)	x	x
Attention (WAIS-IV Forward Digit Span subtest)	x	x
Working memory (WAIS-IV Backward Digit Span subtest)	x	x
Learning/memory (RAVLT)	x	x
Executive function (TMT, SCWT, FAS)	x	x
Processing speed (WAIS-IV Digit Symbol-Coding subtest, SDMT)	x	x
Subjective cognition (PDQ-5)	x	x
Anxiety (GAD-7)	x	x
Depression (PHQ-9)	x	x
PTSD (TOP-8)	x	x
Functionality (WHODAS 2.0)	x	x
HRQoL (SF-12)	x	x

*ICU* Intensive Care Unit, *MoCA* Montreal Cognitive Assessment, *CRQ* Cognitive Reserve Questionnaire, *NART* National Adult Reading Test, *WAIS-IV* Wechsler Adult Intelligence Scale-IV, *RAVLT* Rey Auditory Verbal Learning Test, *TMT* Trail Making Test, *SCWT* Stroop Color and Word Test, *FAS* Verbal phonemic fluency test, *SDMT* Symbol Digit Modalities Test, *PDQ-5* Perceived Deficits Questionnaire-5, *GAD-7* Generalized Anxiety Disorder-7, *PHQ-9* Patient Health Questionnaire-9, *PTSD* Posttraumatic stress disorder, *TOP-8* Treatment-Outcome Post-Traumatic Stress Disorder Scale, *WHODAS 2.0* 12-item World Health Organization Disability Assessment Schedule 2.0, *HRQoL* Health-related quality of life, *SF-12* 12-item Short-Form Health Survey

^a^If not evaluated at 3 months due to non-attendance at the follow-up visit, it will be evaluated at 12 months

#### Cognition

Cognitive reserve will be assessed with the 8-item self-reported Cognitive Reserve Questionnaire (CRQ) (range 0–25) (Cronbach’s alpha = 0.80–0.96) [59, 60] and intelligence quotient (IQ) with the National Adult Reading Test, which provides a reliable estimate of premorbid IQ (*r* = 0.69–0.81) [61, 62]. CRQ and estimated premorbid IQ data will only be collected once at three or 12 months of follow-up.

Objective cognition will be assessed with the MoCA screening test [57, 58] and a comprehensive battery of validated neuropsychological tests including measures of attention (WAIS-IV Forward Digit Span subtest), working memory (WAIS-IV Backward Digit Span subtest), learning/memory (RAVLT), executive function (TMT, SCWT and FAS) and processing speed (WAIS-IV Digit Symbol-Coding subtest and SDMT). Raw data from neuropsychological tests will be standardized using population-based norms [30–35] and combined into a composite index of global cognition (z-score) established on the basis of previous studies [6–8, 13, 14].

Subjective cognition will be assessed with the 5-item self-reported PDQ-5 questionnaire (Cronbach’s alpha = 0.81–0.96) [37, 38].

#### Mental health

Anxiety will be assessed with the 7-item self-reported GAD-7 questionnaire (Cronbach’s alpha = 0.92–0.94) [39, 40], depression with the 9-item self-reported PHQ-9 questionnaire (Cronbach’s alpha = 0.86–0.89) [41, 42] and PTSD with the 8-item hetero-administered TOP-8 scale (Cronbach’s alpha = 0.79) [43, 44].

#### Functionality and HRQoL

Functionality will be assessed with the 12-item self-reported WHODAS 2.0 schedule (Cronbach’s alpha = 0.94–0.98) [45] and HRQoL with the 12-item self-reported SF-12 survey (Cronbach’s alpha = 0.69–0.70) [46, 47].

#### Additional variables

For each cognitive stimulation and relaxation session with the RGS-ICU platform, the start and end time, the protocols performed, the duration of each protocol, the difficulty levels performed (only cognitive protocols), the number of hits and failures per difficulty level (only cognitive protocols), and the number and reasons for interruption and/or early termination of the sessions (if applicable) will be recorded.

The following data will also be collected: days of delirium and invasive MV, days of ICU and hospital stay, discharge destination (home, home hospitalization or social-health center), days to return to work after ICU discharge (working age patients only), number of hospital and ICU readmissions, and need for psychopharmacological treatment, psychotherapy, cognitive rehabilitation and physical rehabilitation after ICU discharge.

These data will be collected at ICU discharge or during post-ICU follow-up visits, as appropriate. If necessary, electronic medical records will be reviewed (Table 4).

**Table 4:** Additional variables collected during and after the ICU stay

	**Day** **1**	**Day** **2**	**Day** **…**	**Day 28/ICU discharge**	**3 months after ICU discharge**	**12 months after ICU discharge**
**Intervention characteristics (for each session)**^a^						
Star and end time	x	x	x	x		
Protocols performed	x	x	x	x		
Duration of each protocol	x	x	x	x		
Difficulty levels performed (only cognitive protocols)	x	x	x	x		
Number of hits and failures per difficulty level (only cognitive protocols)	x	x	x	x		
Number and reason for interruption or early termination (if applicable)	x	x	x	x		
**Other data**						
Days of delirium				x		
Days of invasive MV				x		
Days of ICU stay				x		
Days of hospital stay (including ICU stay)^b^					x	
Discharge destination^b^					x	
Days to return to work after ICU discharge (working age patients only)^c^					x	
Number of hospitals readmissions (excluding ICU readmissions)					x^d^	x^e^
Number of ICU readmissions					x^d^	x^e^
Psychopharmacological treatment^f^					x^d^	x^e^
Psychotherapy					x^d^	x^e^
Cognitive rehabilitation					x^d^	x^e^
Physical rehabilitation					x^d^	x^e^

*ICU* Intensive care unit, *MV* Mechanical ventilation

^a^Data automatically recorded by the RGS-ICU system in a secure cloud database, except for the number and reason for interruption or early termination of sessions, which will be recorded manually

^b^If the patient does not attend the 3-month follow-up visit, these data will be collected at the 12-month follow-up visit

^c^If the patient has not yet returned to work or does not attend the 3-month follow-up visit, this information will be collected at the 12-month follow-up visit

^d^The time elapsed between ICU discharge and the 3-month follow-up visit will be considered

^e^The time elapsed between the 3-month and 12-month follow-up visits will be considered. If the patient does not attend the 3-month follow-up visit, the time elapsed between ICU discharge and the 12-month follow-up visit will be considered

^f^Benzodiazepines, antidepressants, anticonvulsants/mood stabilizers and antipsychotics will be considered

#### Data management and confidentiality

Case Report Forms (CRFs) will be kept in locked file cabinets and all data will be entered in electronic CRFs (eCRFs) in a study-specific REDCap database protected by username and password. Patients will always be identified by a code, so that clinical data concerning them will never be linked to their personal data. In addition, personal and non-personal data will be stored in decoupled REDCap databases to ensure anonymity and confidentiality of the information collected. All records will be pseudo-anonymized and a master file linking the pseudo-anonymized data to patient identifications will be maintained within each center's network. Once this procedure has been carried out, the data will be integrated and stored in a relational database located in the servers of the University of the Balearic Islands-Research Institute of Health Science. From this moment on, aggregated and non-identifiable data will be used, maintaining a variable that is the origin of the data. The data will be securely stored in local servers protected by username and password access and will only be accessible by systems with secure virtual private network connection and secure credentials. All study staff will have access to the data for research purposes, with the obligation to maintain strict confidentiality. Data will be stored for at least 5 years after the end of the trial and then destroyed if no longer needed for research. Each partner will be responsible for data management in its respective center. Harmonization workshops will be organized to ensure that data are collected, assessed, and managed in a homogeneous manner. The staff trained to administer the intervention, collect and assess patient outcomes will be four investigators, two at the Parc Taulí University Hospital and two at the Son Llàtzer University Hospital.

#### Statistical methods

Results will be presented as mean (standard deviation) or frequency (%). Depending on whether the data have a normal distribution, parametric or nonparametric statistics will be used. In case of non-normal data, the median (range) will be reported. To compare results between the RGS-ICU and TAU groups over time, intersubject and intrasubject analyses will be performed, adjusting for multiple comparisons when necessary (e.g., Bonferroni correction). More specifically, intention-to-treat and per protocol analyses will be used. To address missing data, a last observation carried forward analysis together with a complete-case analysis will be performed. Where appropriate, analyses will be controlled for age, sex, delirium, invasive MV, type and dose of pharmacological treatment and/or cognitive reserve (CRQ). Effect sizes will be reported for all significant and non-significant data. Statistical significance will be set at *p* < 0.05. Analyses will be performed using the most recent version of the Statistical Package for the Social Sciences (IBM SPSS Statistics for Windows, Armonk, NY: IBM Corp).

To compare sociodemographic and clinical data between the RGS-ICU and TAU groups, an intersubject analysis will be performed. Intervention characteristics (e.g., session duration, protocols performed, etc.), intervention safety (i.e., number of sessions terminated early due to unsafe events on physiological parameters) and intervention tolerability (e.g., fatigue, extreme sleepiness, confusion) in patients assigned to the RGS-ICU group will be analyzed using descriptive statistics. To compare experienced comfort (PEECE) between the RGS-ICU and TAU groups, an intersubject analysis will be performed. To compare cognition (MoCA, WAIS-IV Forward Digit Span, WAIS-IV Backward Digit Span, RAVLT, TMT, SCWT, FAS, WAIS-IV Digit Symbol-Coding, SDMT and PDQ-5), mental health (GAD-7, PHQ-9 and TOP-8), functionality (WHODAS 2.0) and HRQoL (SF-12) between the RGS-ICU and TAU groups over time, intersubject and intrasubject analyses will be used.

Other analyses will include comparison of changes in the intensity of dyspnea, pain, worry and sadness (VAS) before and after each session in patients assigned to the RGS-ICU group. To this aim, an intrasubject analysis will be used.

#### Data monitoring

A study monitor who will not be involved in patient care, intervention, data collection and assessment, or study coordination will oversee all study procedures in accordance with Good Clinical Practice guidelines. The study monitor will also ensure that written informed consents have been obtained, that (e)CRFs have been created correctly (e.g., including ranges to avoid exceeding minimum and maximum values, including additional fields to add information to ensure proper recording of data), and that the data are stored securely in locked file cabinets and/or in the REDCap database. The study monitor will also assess the completeness and correctness of the data twice a year and provide technical support to other study staff.

#### Harms and adverse event reporting

Previous studies have shown that VR-based cognitive and relaxation interventions can be used safely in the ICU, even in patients with invasive MV [13, 19, 20]. Adverse events (e.g., unsafe physiological parameters, unintentional removal of catheters, endotracheal tubes, tracheostomy cannulas), whether or not related to the use of the RGS-ICU platform, will be collected and analyzed for all participants. No reason for interruption or suspension of the trial is foreseen. However, following the European Commission guidelines on the collection, verification and reporting of adverse events arising from clinical trials [63], in the event of a (serious) adverse event that poses a risk to participants or is causally related to the use of the RGS-ICU platform, the principal investigator may interrupt and/or suspend the trial, and the ethics committee and other relevant parties will be informed.

#### Auditing and protocol amendment

The study staff of each participating center will be responsible for complying with the study protocol and for reporting any deviation or request for modifications to the study coordinator and monitor and the principal investigator. All decisions regarding study procedures and protocol modifications will be made with consensus of the four partners, communicated to relevant parties and ethics committee for approval prior to implementation, and posted on Clinicaltrials.gov. However, in emergency circumstances, deviations from the study protocol may be made to protect the rights, safety and welfare of human subjects without prior ethics committee approval. Any deviation from the study protocol will be fully documented in the (e)CRF. For quality assurance, the study coordinator and monitor, principal investigator and ethics committee may have access to the source data and all study-related records, with the obligation to maintain strict confidentiality.

## Ethics and dissemination

### Consent or assent

The clinical staff in charge of patient screening and recruitment at each hospital will be responsible for obtaining written informed consent from the potential candidates and/or authorized representatives. The consent form contains all the information about the intervention and the data collected and evaluated and informs patients that they can exercise their right of access, modification, opposition and cancellation at any time. Patients are also informed that they may withdraw from the study at any time but that the data collected so far may continue to be used within the framework of the objectives of this trial unless they expressly object. Likewise, patients will be informed that they may be withdrawn from the study if deemed appropriate by the principal investigator, either for safety reasons, due to an adverse event derived from the intervention under study or due to non-compliance with the established procedures. The patient will receive an adequate explanation of the reason for the withdrawal of the study and will always continue to receive the standard medical treatment for his/her pathology. Finally, patients will be informed that their data may be shared with third parties (e.g., other research centers and universities), other countries, and the relevant regulatory authorities, while respecting the anonymity and confidentiality of the data, and within the framework of the objectives of this study. This trial does not involve collecting biological specimens.

### Dissemination policy and access to data

The progress of the project will be regularly disseminated in the media and networks. Interim analyses will only be conducted to inform funders of the progress of the study, but no results will be published before the end of the trial, scheduled for summer 2026. Once the study is completed, results will be submitted for publication in peer-reviewed journals, presented at regional, national, and international scientific meetings, and disseminated to the public and other interested parties. At present, no publication restrictions are foreseen. The datasets and statistical code will be available upon reasonable request to the corresponding author(s) once the results have been published, as are the full protocol and informed consent materials.

## Discussion

This is a two-arm, parallel-group, superiority RCT aimed at evaluating the efficacy and safety of receiving early cognitive stimulation and relaxation as an adjunct to standard ICU care compared to receiving standard ICU care alone. This trial responds to the need to humanize intensive care [64], a new paradigm of human-centered ICU care that aims to actively involve patients in their recovery process, as intended by the RGS-ICU intervention.

This RCT builds on previous PoC and clinical pilots that demonstrated that neuropsychological e-health interventions based on VR techniques, whether immersive or not, used as an adjunct to standard ICU care, in addition to being safe for patients with critical illness [19], can help reduce anxiety during the ICU stay [20], as well as improve working memory performance after ICU discharge [13]. However, these studies were limited by methodological issues such as single-center designs, small sample sizes (n ≤ 21 subjects per group), heterogeneous diagnoses, interventions that included cognitive but not relaxation protocols, and absence of assessment of variables such as experienced comfort, subjective cognition, functionality and HRQoL.

In an attempt to overcome these limitations, the RGS-ICU trial has been designed, whose main strengths include a multicenter design, strict inclusion/exclusion criteria, an adequately powered sample size to test the primary outcome (i.e., objective cognition 12 months after ICU discharge), the use of an enhanced and optimized e-health intervention combining cognitive protocols to stimulate the brain with relaxation videos to promote stress and anxiety reduction, and the administration of a full neuropsychological battery to characterize the cognitive, mental health, functional and HRQoL profile of survivors of critical illness. This trial will also benefit from monitoring patients' physiological parameters as part of standard ICU care to ensure the safety of the intervention and that it is adapted to their mental status and mobility level in real time. In contrast, its open design is a limitation that will be compensated by blinding the data analysis by an independent statistician. Following SPIRIT guidelines for reporting study protocols [22], the sample size has been calculated for the primary outcome, so the study may be underpowered for test other outcomes. The use of self-reported measures to assess mental health may also be a limitation of the study.

By relaxing patients and increasing brain activity in areas related to cognition and emotion, as demonstrated in previous PoC and clinical pilots using a surrogate measure of cognitive function such as HRV [13, 19, 20], the RGS-ICU intervention could help preserve neuropsychological functioning in survivors of critical illness, especially in patients who are mentally vulnerable, such as elderly patients, patients with delirium or patients undergoing invasive MV. Thus, the results of the present trial could contribute to establishing a new paradigm of supportive care adjuvant to standard ICU care (and perhaps even superior to standard ICU care alone) to improve neuropsychological outcomes during and after the ICU stay. In addition to the clinical impact, the results could also have a relevant socioeconomic impact by accelerating the recovery process. In fact, interventions similar to RGS-ICU but focused on physical and functional rehabilitation to prevent ICU-acquired muscle weakness have shown a reduction of ICU admission of up to 1.4 days [65].

Given that the RGS-ICU platform interprets the physical movements of patients' upper extremities to interact with cognitive multimedia content, it is anticipated that the results of the present trial may also encourage the introduction of rehabilitation protocols that combine physical and cognitive exercises with relaxing videos in a single technological tool that addresses all three dimensions of PICS at once. Future trials should also consider extending early cognitive stimulation and relaxation beyond ICU stay to contribute to the rehabilitation and recovery process of survivors of critical illness affected by PICS.

## Supplementary Information

Additional file 1. Fulfilled SPIRIT checklist for reporting study protocols. SPIRIT, Standard Protocol Items: Recommendations for Interventional Trials.

Additional file 2. More images of the RGS-ICU platform in a real operating environment. RGS-ICU, Rehabilitation Gaming System for Intensive Care Units.

Additional file 3. Video with examples of the gamified cognitive protocols of the RGS-ICU platform recorded in a real operating environment. RGS-ICU, Rehabilitation Gaming System for Intensive Care Units.

Additional file 4. Data on usability and satisfaction with the RGS-ICU platform from a group of 15 patients with critical illness. RGS-ICU, Rehabilitation Gaming System for Intensive Care Units.

## Data Availability

The datasets used and/or analyzed during the current study are available from the corresponding authors, S.F.G. (msfernandez@tauli.cat) and G.N.V. (g.navarra@tauli.cat), on reasonable request.

## Abbreviations

APACHE II: Acute Physiology and Chronic Health Evaluation II
CAM-ICU-7: Confusion Assessment Method for the Intensive Care Unit-7
CCI: Charlson Comorbidity Index
Covid-19: Coronavirus disease 2019
CRF: Case report form
CRQ: Cognitive Reserve Questionnaire
eCRF: Electronic case report form
ENRIC: Early Neurocognitive Rehabilitation in Intensive Care
FAS: Phonemic verbal fluency
GAD-7: Generalized Anxiety Disorder-7
GCS: Glasgow Coma Scale
HR: Heart rate
HRQoL: Health-related quality of life
HRV: Heart rate variability
ICU: Intensive care unit
IQ: Intelligence quotient
MoCA: Montreal Cognitive Assessment
MV: Mechanical ventilation
PDQ-5: Perceived Deficits Questionaire-5
PEECE: Patient Evaluation of Emotional Comfort Experienced
PHQ-9: Patient Health Questionnaire-9
PoC: Proof of concept
PTSD: Post-traumatic stress disorder
RASS: Richmond Agitation-Sedation Scale
RAVLT: Rey Auditory Verbal Learning Test
RCT: Randomized clinical trial
REDCap: Research Electronic Data Capture
RGS-ICU: Rehabilitation Gaming System for Intensive Care Units
RR: Respiratory rate
SAPS 3: Simplified Acute Physiology Score 3
SCWT: Stroop Color and Word Test
SDMT: Symbol Digit Modalities Test
SF-12: 12-Item Short-Form Health Survey
SOFA: Sequential Organ Failure Assessment
SPIRIT: The Standard Protocol Items: Recommendations for Interventional Trials
SpO2: Peripheral oxygen saturation
TAU: Treatment as usual
TMT: Trail Making Test
TOP-8: Treatment-Outcome Post-Traumatic Stress Disorder Scale
TV: Television
VAS: Visual analogue scale
VR: Virtual reality
WAIS-IV: Wechsler Adult Intelligence Scale-IV
WHODAS 2.0: 12-Item World Health Organization Disability Assessment Schedule 2.0
