# Supplementary material for: Efficacy and safety of a non-immersive virtual reality-based neuropsychological intervention for cognitive stimulation and relaxation in patients with critical illness: study protocol of a randomized clinical trial (RGS-ICU)
Source: BMC Psychiatry. 2024 Dec 18;24:917. doi: 10.1186/s12888-024-06360-4 (PMC11654385; doi:10.1186/s12888-024-06360-4)
Supplement: Supplementary file 2 — Additional file 2. More images of the RGS-ICU platform in a real operating environment. RGS-ICU, Rehabilitation Gaming System for Intensive Care Units. [file 12888_2024_6360_MOESM2_ESM.pdf]

RGSICU

Rehabilitation Gaming System for Intensive Care Units

UIB Universitat de les Illes Balears

Parc Taulí
Institut d'Investigació i Innovació I3PT

eodyne

Fundació La Marató 3cat

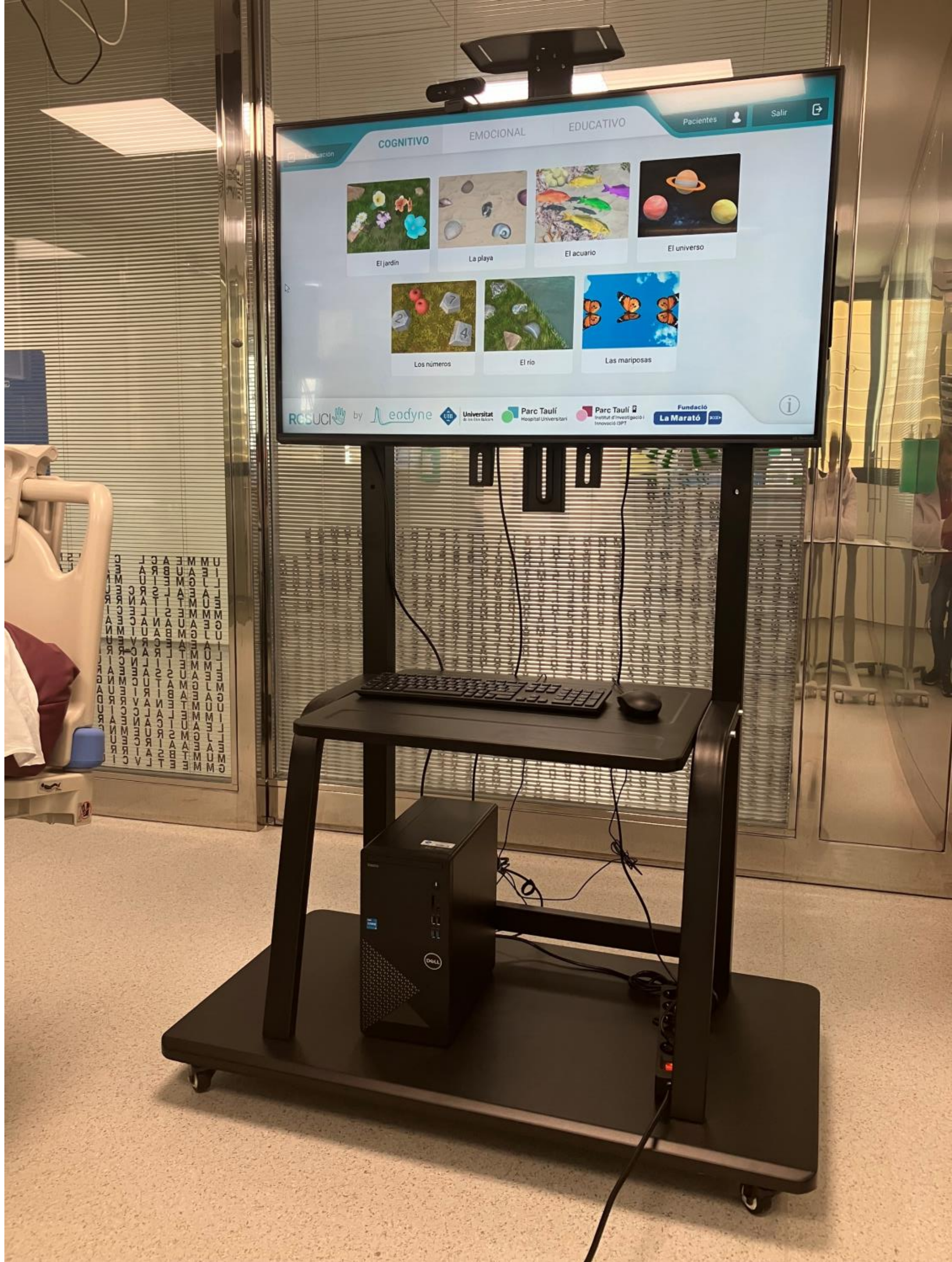

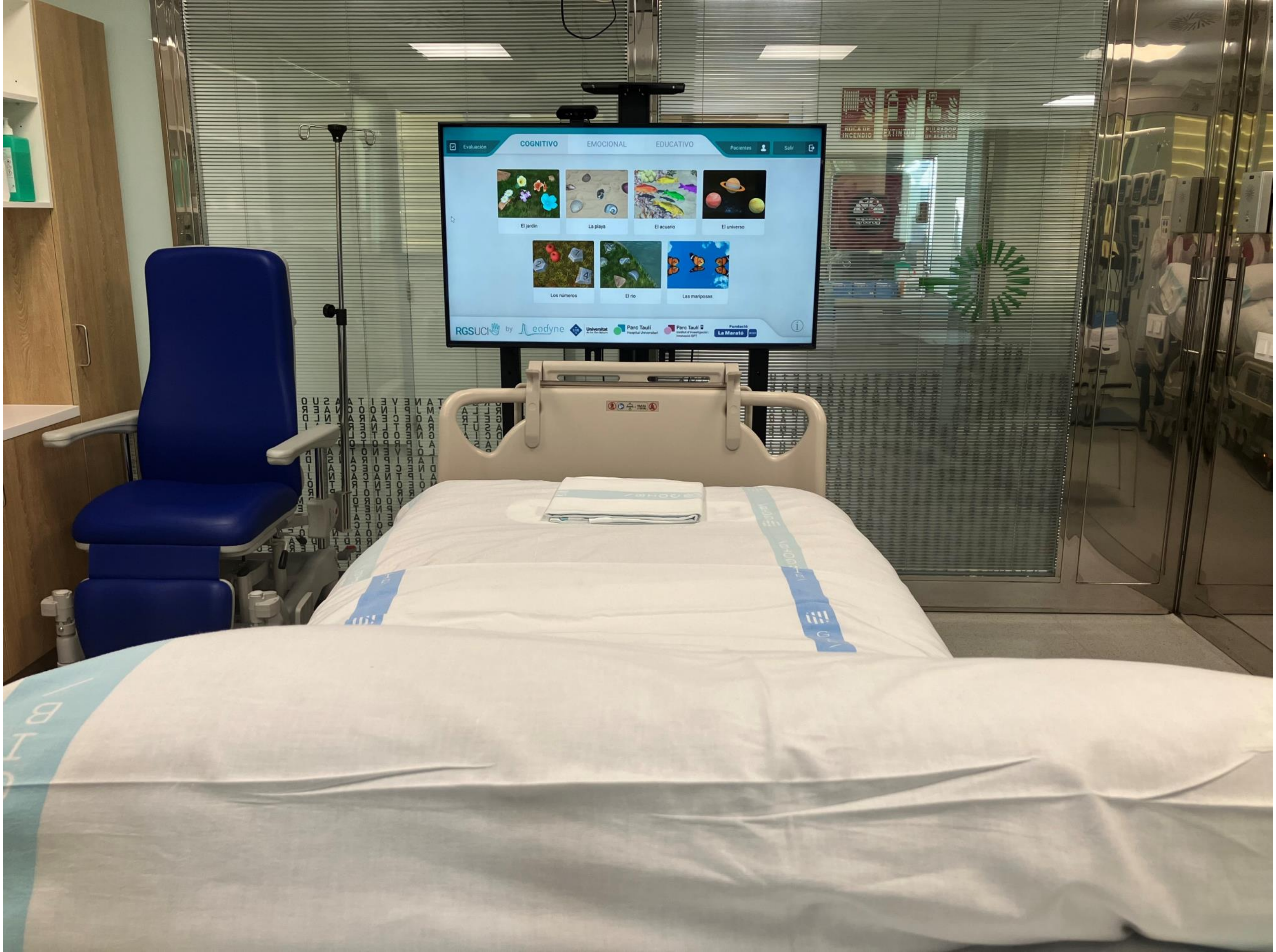
Evaluación
COGNITIVO
EMOCIONAL
EDUCATIVO
Pacientes
Salir
El jardín
La playa
El acuario
El universo
Los números
El río
Las mariposas
RGSUCI
by
eodyne
Parc Taulí
La Marató

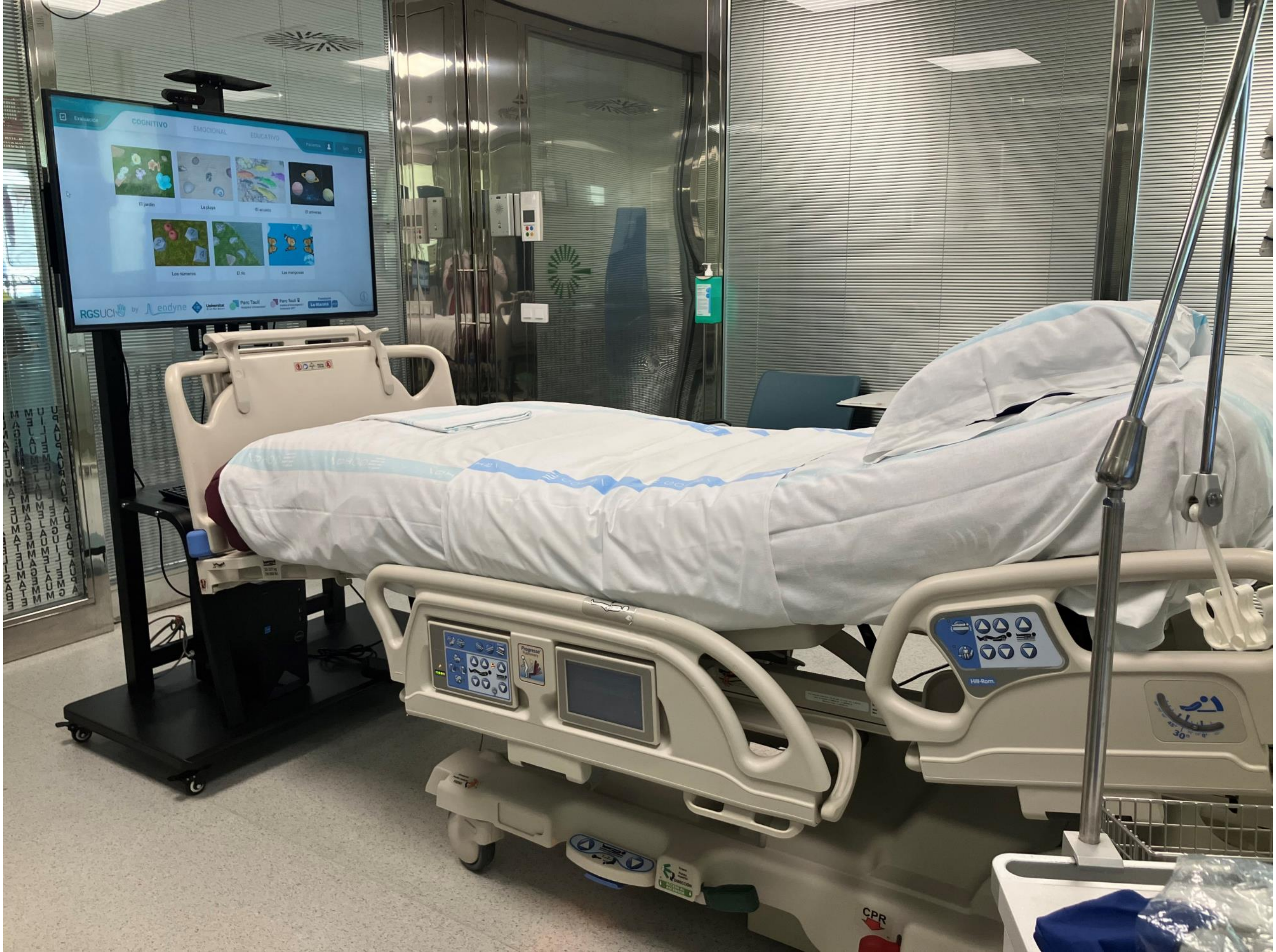
COGNITIVO
EMOCIONAL
EDUCATIVO
RGSUCI
by
eodyne
Hill-Rom
CPR

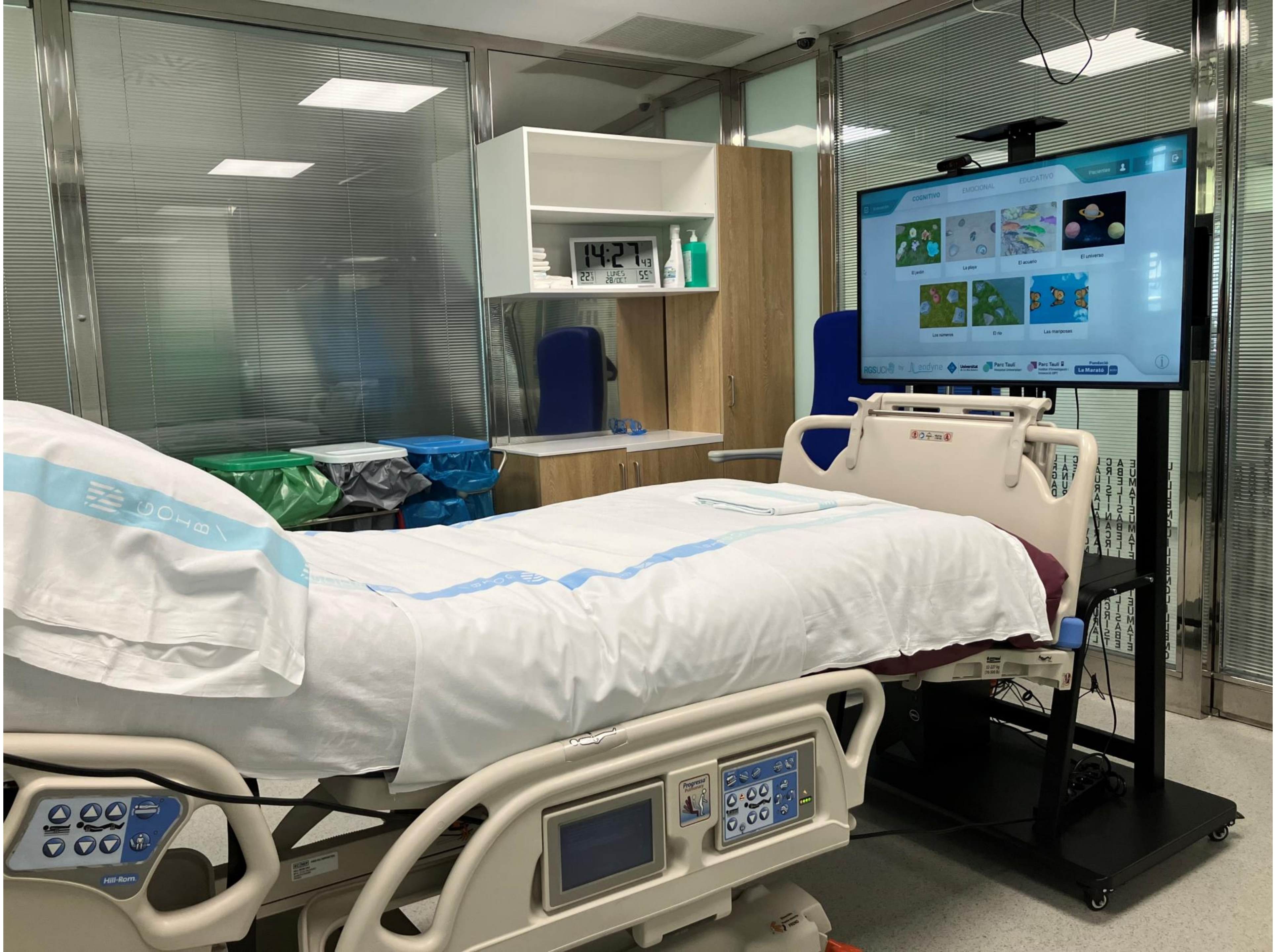
COGNITIVO
EMOCIONAL
EDUCATIVO
Pacientes
El jardín
La playa
El acuario
El universo
Los números
El río
Las mariposas
RGSUCI
by
eodyne
Universitat
Parc Taulí
Parc Taulí
Fundació
La Marató
14:27
LUNES
28/OCT
Hill-Rom
Progressa

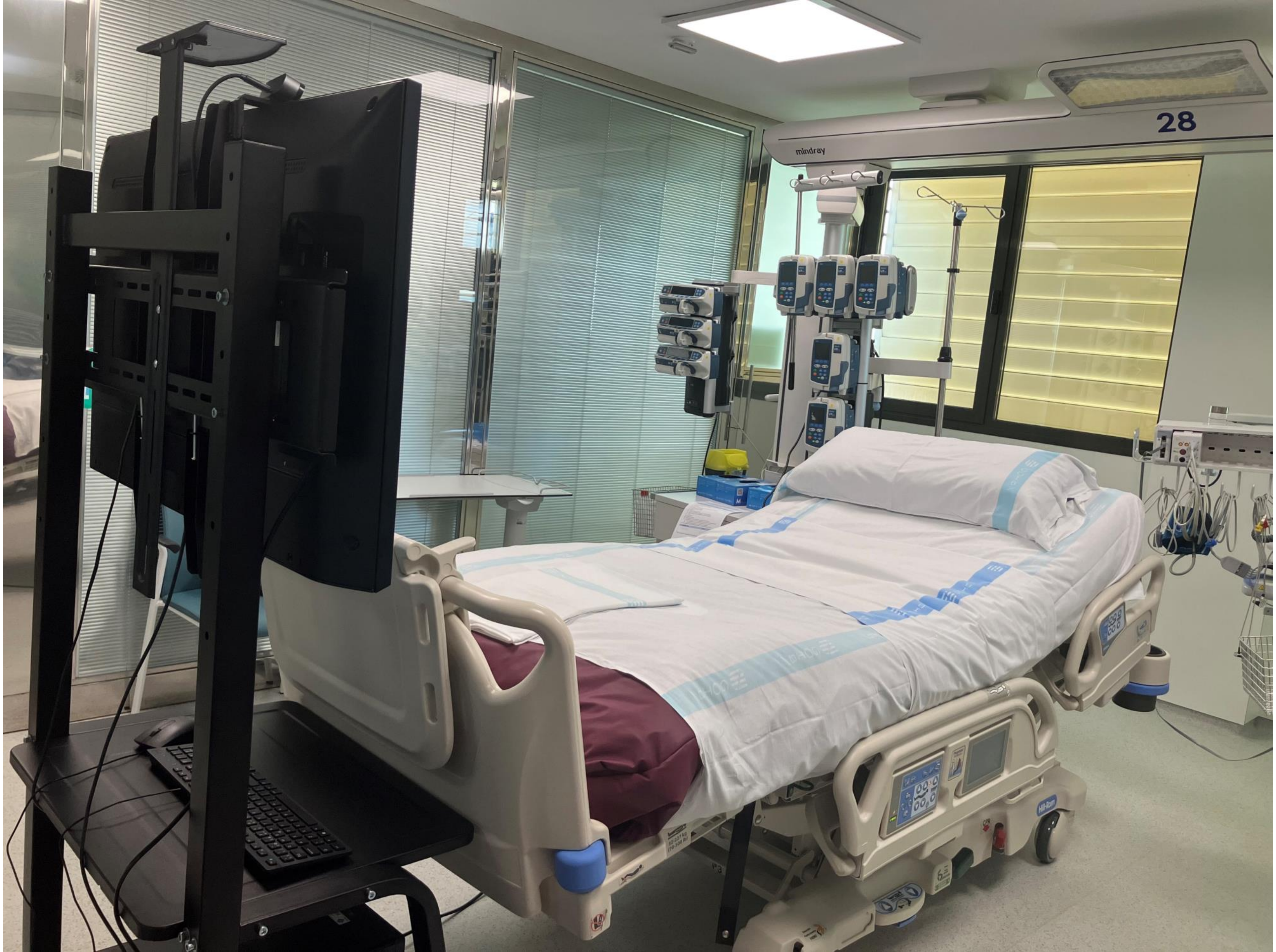
28
mindray

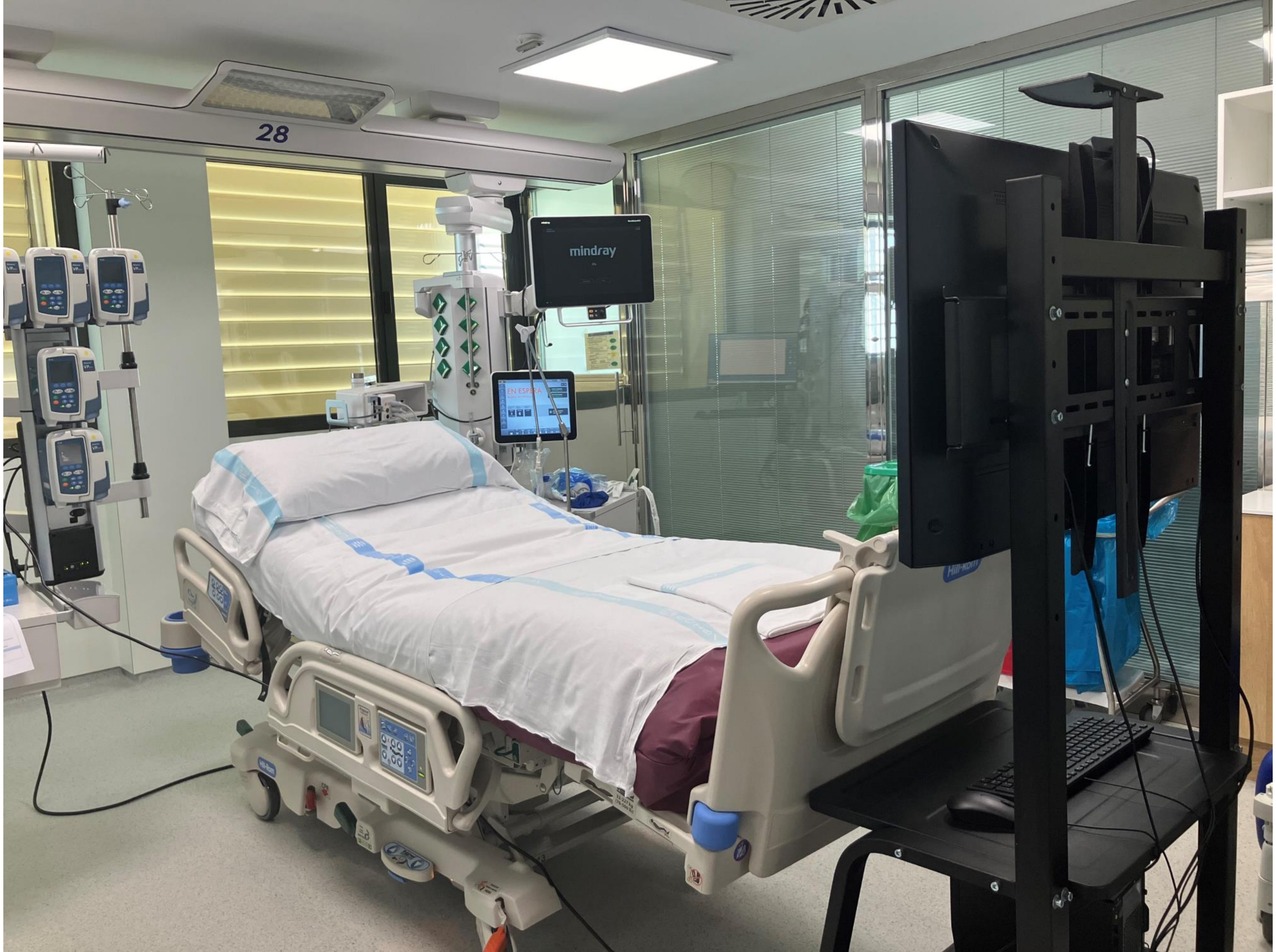
28
mindray
EN ESPERA
