# Supplementary material for: Efficacy and safety of a non-immersive virtual reality-based neuropsychological intervention for cognitive stimulation and relaxation in patients with critical illness: study protocol of a randomized clinical trial (RGS-ICU)
Source: BMC Psychiatry. 2024 Dec 18;24:917. doi: 10.1186/s12888-024-06360-4 (PMC11654385; doi:10.1186/s12888-024-06360-4)
Supplement: Supplementary file 4 — Additional file 4. Data on usability and satisfaction with the RGS-ICU platform from a group of 15 patients with critical illness. RGS-ICU, Rehabilitation Gaming System for Intensive Care Units. [file 12888_2024_6360_MOESM4_ESM.pdf]

Additional file 4

Study on usability and satisfaction with the RGS-ICU platform

The primary objective of this study was to evaluate the ease of use, overall difficulty, and patient satisfaction with the cognitive protocols included in the RGS-ICU platform in a real operating environment. Secondary objectives were to perform a technical and functional test of the Rehabilitation Gaming System for Intensive Care Units (RGS-ICU) platform, including the motion capture imager, TV screen, and sound volume.

For this purpose, 15 patients with critical illness ≥18 years old, with a planned intensive care unit (ICU) admission ≥24 hours and no history of psychiatric, neurological or brain damage were included. Patients who were too ill to participate or who did not consent were excluded.

Patients tested the cognitive protocols of the RGS-ICU platform at least once during their ICU admission when they were mentally competent, defined as a score ≥13 on the Glasgow Coma Scale, a score between -1 and +1 on the Richmond Agitation-Sedation Scale, and a score ≤2 on the Confusion Assessment Method for the ICU-7.

Patients rated the technical and functional performance, ease of use, overall difficulty, and satisfaction with the RGS-ICU platform and cognitive protocols on the last day they tested it during their ICU stay by means of several multiple-choice questions developed by the research team itself.

Data were collected between July and September 2021 in the ICU of the Parc Taulí University Hospital of Sabadell, Barcelona, Catalonia (Spain).

Data were analyzed using descriptive statistics. Analyses were performed with the Statistical Package for the Social Sciences software.

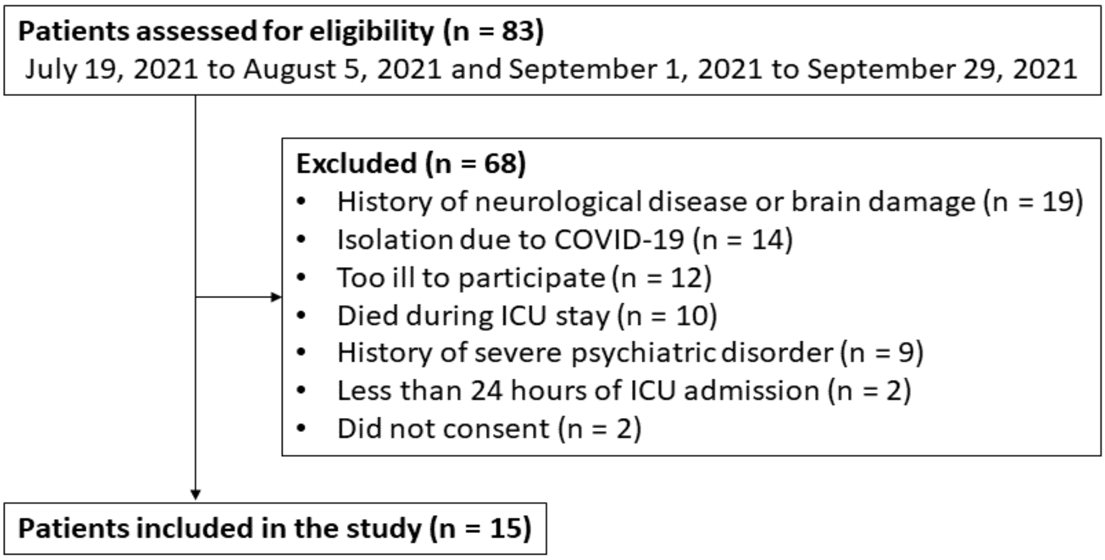

Figure 1. Flow diagram of the study. COVID-19, coronavirus disease 2019; ICU, intensive care unit.

Table 1. Sociodemographic and clinical characteristics of patients

Median [min-max] is reported unless otherwise specified	
N	15
Age, years	67.4 [21.9-84.7]
Female sex, n (%)	6 (40)
Diagnosis, n (%)	
Medical	13 (86.7)
Surgical	2 (13.3)
Length of ICU stay, days	7 [1-46]
Length of hospital stay, days	27 [5-75]
Need for mechanical ventilation, n (%)	10 (66.7)
Invasive	6 (60)
Non-invasive	4 (40)
Duration of invasive mechanical ventilation, days	5.5 [2-35]
Presence of delirium (≥1 day(s)), n (%)	1 (6.7)

Table 2. Characteristics of the proof-of-concept study

Median [min-max] is reported unless otherwise specified	
Number of sessions, n	22
Number of sessions per patient	1 [1-3]
Number of cognitive protocols, n	43
Protocol 1: bees (attention), n (%)	13 (30.2)
Protocol 2: beach (learning/memory), n (%)	11 (25.6)
Protocol 3: fishing (executive functions), n (%)	15 (34.9)
Protocol 4: planets (working memory), n (%)	4 (9.3)
Number of cognitive protocols per session*	2 [0-3]
Number of completed sessions, n (%)	11 (50)
Reason for interruption of sessions, n (%)	

Fatigue	8 (72.7)
Pain	1 (9.1)
Technical problem	2 (18.2)
Duration of sessions, minutes**	9.9 [4.3-24.5]
Position during the sessions, n (%)	
Bedridden	17 (73.9)
Seated	6 (26.1)

* Two sessions were interrupted due to technical problems and no cognitive protocol could be administered. ** Calculated over a total of 20 sessions, since the two sessions that were interrupted due to technical problems have been discarded as it was not possible to administer any cognitive protocol

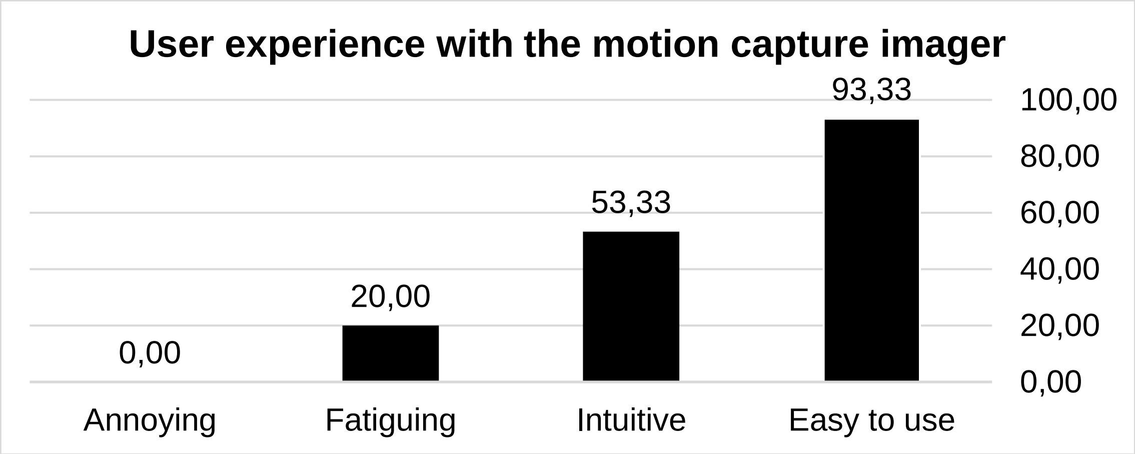

Figure 2. User experience with the motion capture imager (% are reported)

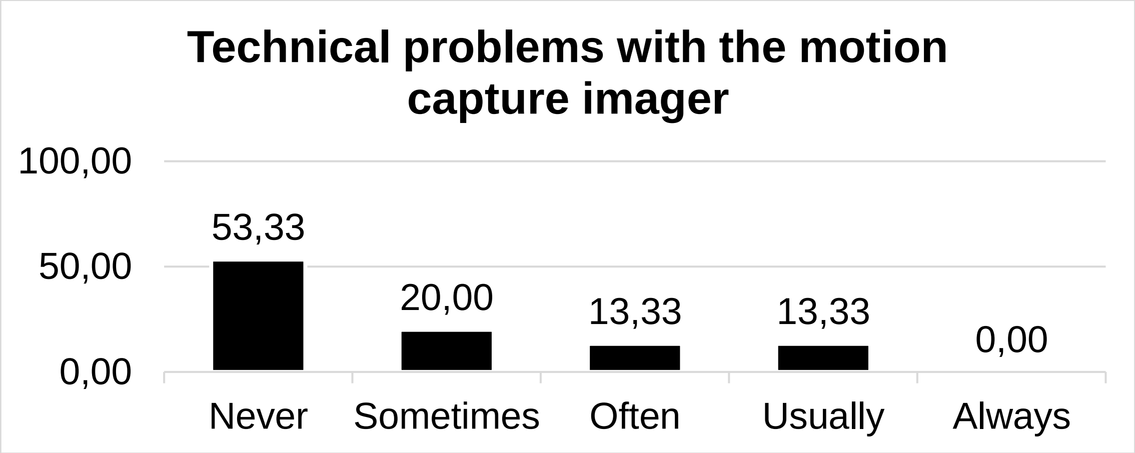

Figure 3. Frequency (%) of technical problems with the motion capture imager

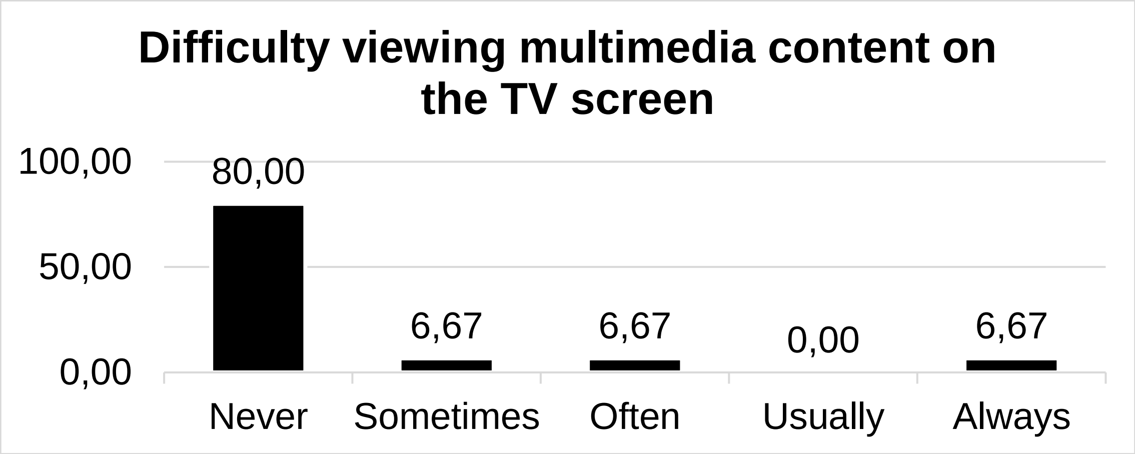

Figure 4. Frequency (%) of difficulties in viewing multimedia content on the TV screen (e.g., light reflection, screen brightness, size and shape of stimuli)

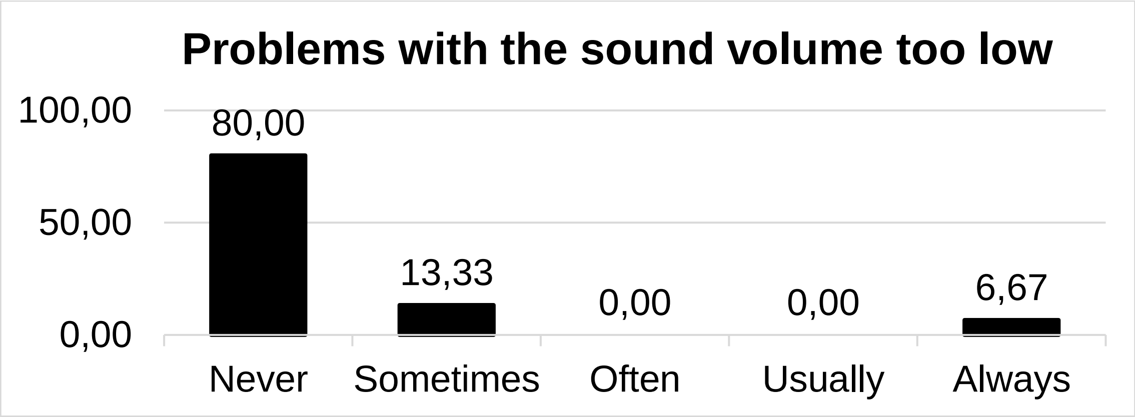

Figure 5. Frequency (%) of problems with the sound volume too low

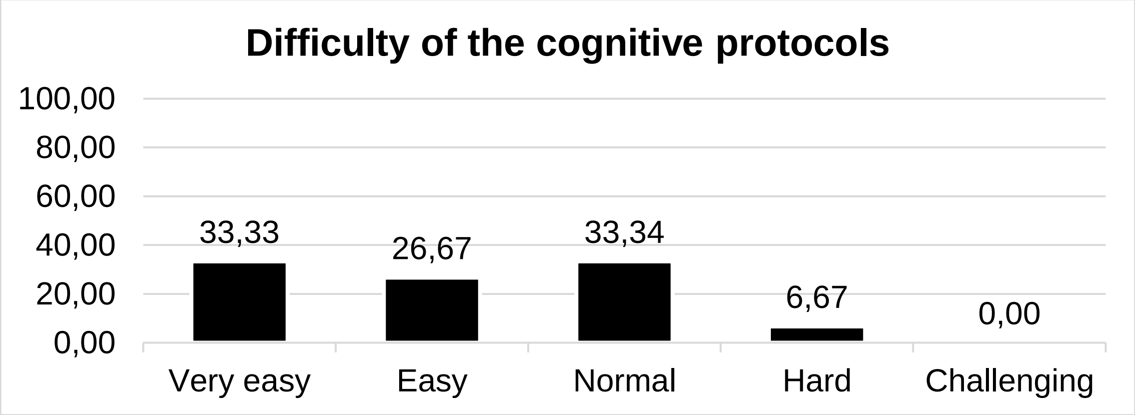

Figure 6. Difficulty of the cognitive protocols (% are reported)

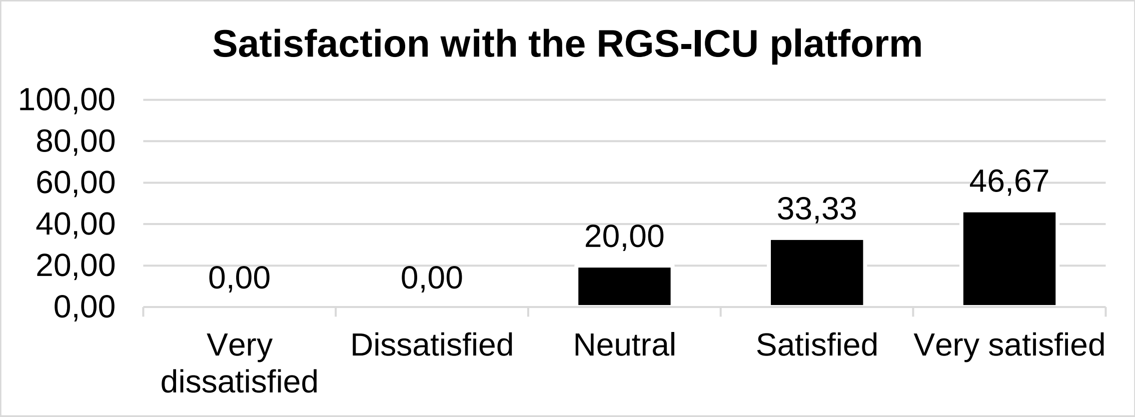

Figure 7. Satisfaction with the RGS-ICU platform (% are reported). Overall satisfaction measured with a Visual Analogue Scale ranging from 0 (minimum) to 5 (maximum) was 4.3 points.

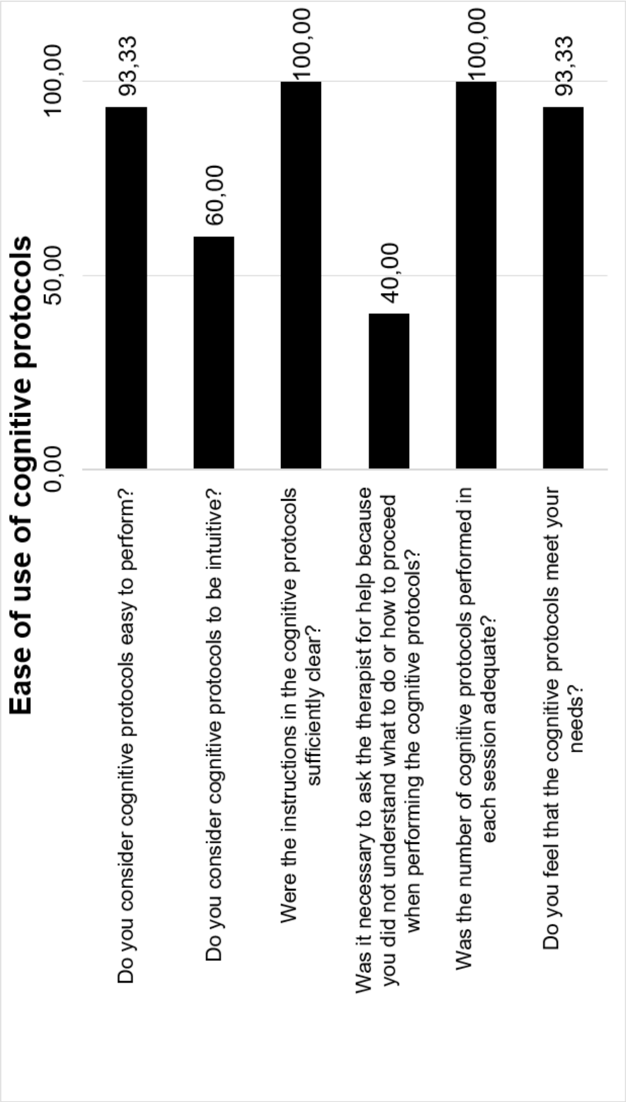

Figure 8. Ease of use of cognitive protocols (% are reported)

These data were used to optimize the technical and functional performance of the RGS-ICU platform and the usability of the cognitive protocols and difficulty levels to better adapt the intervention to the needs of patients with critical illness.

All procedures contributing to this work comply with the ethical standards of the relevant national and institutional committees on human experimentation and with the Helsinki Declaration of 1975, as revised in 2008. Ethical approval has been provided by the Ethics Committee of the Parc Taulí University Hospital of Sabadell, Barcelona, Catalonia (Spain) (#2021/3011). Written informed consent was obtained from all participants.

This project was funded by ACCIO Nuclis d'R+D empresarial, project number RD17-1-0015; CIBER -Consorcio Centro de Investigación Biomédica en Red- CB06/06/1097, Instituto de Salud Carlos III, Ministerio de Ciencia e Innovación and Unión Europea - European Regional Development Fund; CERCA Programme/Generalitat de Catalunya; and Institut d'Investigació i Innovació Parc Taulí-I3PT. The funders played no role in study design, data collection and analysis, manuscript preparation, or decision to publish.
